# Visual adaptation of opsin genes to the aquatic environment in sea snakes

**DOI:** 10.1186/s12862-020-01725-1

**Published:** 2020-11-26

**Authors:** Takashi Seiko, Takushi Kishida, Mina Toyama, Takahiko Hariyama, Takashi Okitsu, Akimori Wada, Mamoru Toda, Yoko Satta, Yohey Terai

**Affiliations:** 1 Department of Evolutionary Studies of Biosystems, SOKENDAI (The Graduate University for Advanced Studies), Shonan Village, Hayama, Kanagawa 240-0193 Japan; 2 Wildlife Research Center, Kyoto University, 2-24 Tanaka Sekiden-cho, Sakyo, Kyoto 606-8203 Japan; 3 Department of Biology, Faculty of Medicine, Hamamatsu University School of Medicine, Handayama, Hamamatsu Japan; 4 Laboratory of Organic Chemistry for Life Science, Kobe Pharmaceutical University, 4-19-1, Motoyamakita, Higashinada, Kobe, 658-8558 Japan; 5 Tropical Biosphere Research Center, University of the Ryukyus, Nishihara, Okinawa 903-0213 Japan

**Keywords:** Visual adaptation, Opsin genes, Sea snakes, Visual pigments, Aquatic amniotes

## Abstract

**Background:**

Evolutionary transitions from terrestrial to aquatic life history cause drastic changes in sensory systems. Indeed, the drastic changes in vision have been reported in many aquatic amniotes, convergently. Recently, the opsin genes of the full-aquatic sea snakes have been reported. However, those of the amphibious sea snakes have not been examined in detail.

**Results:**

Here, we investigated opsin genes and visual pigments of sea snakes. We determined the sequences of *SWS1*, *LWS*, and *RH1* genes from one terrestrial, three amphibious and four fully-aquatic elapids. Amino acid replacements at four and one spectra-tuning positions were found in LWS and RH1, respectively. We measured or predicted absorption of LWS and RH1 pigments with A1-derived retinal. During their evolution, blue shifts of LWS pigments have occurred stepwise in amphibious sea snakes and convergently in both amphibious and fully-aquatic species.

**Conclusions:**

Blue shifted LWS pigments may have adapted to deep water or open water environments dominated by blue light. The evolution of opsins differs between marine mammals (cetaceans and pinnipeds) and sea snakes in two fundamental ways: (1) pseudogenization of opsins in marine mammals; and (2) large blue shifts of LWS pigments in sea snakes. It may be possible to explain these two differences at the level of photoreceptor cell composition given that cone and rod cells both exist in mammals whereas only cone cells exist in fully-aquatic sea snakes. We hypothesize that the differences in photoreceptor cell compositions may have differentially affected the evolution of opsins in divergent amniote lineages.

## Background

Among amniotes, several major extant taxonomic groups have returned to the sea and have adapted to an aquatic environment. The best-known group is cetacea, composed of toothed and baleen whales. Sirenia is a group that includes the dugong and manatee which both inhabit relatively shallow water. Among reptiles, hydrophiin sea snakes have also adapted to an aquatic environment completely, and do not rely on terrestrial habitat anymore [1, 2]. Species in these three amniote groups are fully aquatic, give birth to live young, and spend their whole life in water.

Sea snakes are composed of species in the fully aquatic hydrophiins and amphibious laticaudins (also called sea kraits) [3]. Molecular phylogenetic studies have reported that Hydrophiini forms a monophyletic clade with terrestrial snake species, and this clade forms a sister clade with the amphibious Laticaudini [4, 5]. Unlike Hydrophiini, species in Laticaudini come onto the land at night and when females lay eggs [6]. The divergence time between Hydrophiini and Laticaudini is estimated to be approximately 12–20 million years (Ma) [2, 4, 7]. Each of these two taxa are monophyletic and have independently adapted to the aquatic environment [4, 5]. The morphology and the physical features of sea snakes such as a paddle-shaped tail and cutaneous respiration are distinct from terrestrial snakes because of adaptation to the marine environment [1, 8].

Water affects light transmission by scattering and absorption, thereby creating different light conditions between terrestrial and marine environments. Light in shallow water contains a wide range of the visual spectrum, similar to that of terrestrial light, whereas light in deep water is dominated by a narrow band of short wavelengths of light centered around 475 nm [9]. Amniotes dive to different and thereby encounter different light environments, and these differences have affected the evolution of the visual system in marine amniotes.

Visual adaptation provides a prime example of molecular evolution due to natural selection [10]. Vertebrate visual pigments consist of a light-absorbing component (the chromophore), and a protein moiety named opsin [11]. Spectral sensitivity is determined by the chromophore with 11-cis retinal (A1-) or 11-cis 3-dehydroretinal (A2-) derived retinal and by the interaction of the chromophore with the amino acid residues that coat the retinal-binding pocket of the opsin in which the chromophore lies [12]. The replacement of A1- with A2-derived retinal shifts the absorption to a longer wavelength [13, 14]. Both chromophores have been reported in the eyes of reptiles [15, 16]. In vertebrates, several spectral tuning positions in the amino acid sequence of opsins have been reported. Using these sites, absorption spectra of visual pigments have been predicted from opsin gene sequences [12, 17–21].

Vertebrates possess two types of photoreceptor cells with structural and functional differences, cone cells that are active in bright light and contain cone visual pigments (SWS1, SWS2, RH2, LWS) for color vision, and rod cells that contains RH1 pigments for scotopic vision [10, 12, 22]. However, the photoreceptor cells in reptiles differ from this pattern. Reptilian retinas are unique in having multiple photoreceptor cell compositions [23] such as rod and cone [24], all-rod [25], and all-cone [26] types of retina. This multiple composition is explained by a theory of transmutation; the evolutionary transformation of photoreceptors from rod to cone type, or vice versa [27]. Indeed, cone opsins are expressed in the all-rod retina of nocturnal geckos [25, 28], and RH1 is expressed in the all-cone retina of diurnal snakes [29, 30]. The most recent common ancestor (MRCA) of snakes probably possessed a typical rod and cone type of retina [31], and transmutations from rod type to cone and vice versa have occurred multiple times in the evolution of snakes [32]. An all-cone retina has also reported from Hydrophiini [33, 34].

The MRCA of lizards and snakes possessed visual opsins sensitive to short (SWS1 and SWS2), middle (RH2), and long wavelength (LWS) light for color vision, and RH1 for scotopic vision [35, 36]. However, snakes lost two cone opsins (SWS2 and RH2) in their ancestors [35, 36]. As a result, extant species, except fossorial species, express only SWS1, LWS, and RH1 [37]. The MCRA of eutherians also lost the same color opsins, and the MRCAs of cetaceans and pinnipeds (walruses, sea lions and seals) possessed SWS1, LWS and RH1 [10].

Recently, the evolution and diversification of these three opsins has been reported from Hydrophiini. In SWS1, polymorphism for phenylalanine and tyrosine at position 86 (corresponding to position 85 in bovine RH1) is thought to underlie spectral tuning in this opsin that has persisted across Hydrophiini species [38]. This polymorphism may confer both UV and blue sensitivities to heterozygotes individual [38].

The transitions from terrestrial to the marine environment have affected the evolution of opsins in marine mammals. The correlation between foraging depth and spectral sensitivity of the visual pigments was firstly reported in cetacean species [39]. The absorption of the visual pigments from deep-diving species have shifted toward blue compared to shallow-diving species [39]. Subsequently, the molecular mechanism underlying the spectral tuning of both the RH1 and LWS pigments from cetacean species was reported [40, 41]. Shallow diving species have a RH1 with a peak absorbance (λmax) similar to that of terrestrial mammals (500 nm) and possess the aspartic acid, alanine, and serine at position 83, 292, and 299, respectively (represented as 83D, 292A, and 299S), while deep-diving species possess the combination of 83 N, 292S, and 299A with λmax around 479 nm [42, 43]. The correlation between foraging depth and spectral sensitivity of RH1 pigments was also reported in pinnipeds. A deep-diving elephant seal possesses 292S with λmax 483 nm, while the other shallow-diving pinnipeds possess 292A with λmax similar to that of terrestrial mammals [44, 45]. For LWS, the three toothed whale species possesses 292S to give a blue-shifted λmax (522–531 nm) compared to terrestrial mammals (around 560 nm) [40, 46].

In addition to the spectra shifts, opsin genes have been lost during the adaptation to the marine environment in marine mammals. Behavioral experiments showed that the bottlenose dolphin lacks the ability to discriminate color (e.g. color blind) [47]. Indeed, all cetacean species lost the *SWS1* gene [41, 48–50] at the early stage of their evolutionary transition [51] and are color blind. All pinnipeds species also lost the *SWS1* gene [45, 46, 48, 52], indicating convergent degenerations in cetaceans and pinnipeds during their transitions. Furthermore, cetaceans have independently lost the *LWS* multiple times in both toothed and baleen whale lineages [51]. As a consequence, rod monochromatic species have evolved in these two lineages.

Although the impacts of the transitions from land to sea on the visual system of marine mammals (cetaceans and pinnipeds) and Hydrophiini species have been reported previously, the opsins of Laticaudini species have not been studied in detail. In this study, we investigated the evolution of opsins in sea snakes including both amphibious and fully aquatic species to uncover the adaptive process of this group and to compare the evolutionary processes of opsins between divergent amniote lineages.

## Results

### Absorption spectra tuning positions in the opsin gene sequences from sea snakes

**Fig. 1:**
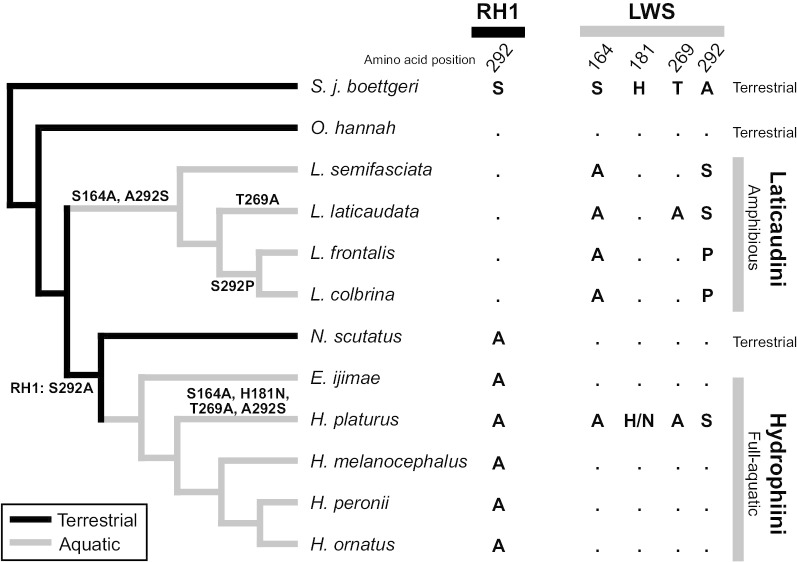
The amino acid replacements at spectra tuning positions of RH1 (left) and LWS (right) among the species analyzed in the study. Amino acids identical to the top line are indicated by dots. The amino acid replacements shown on or under the branches. Amino acid replacement in RH1 is shown as “RH1: S292A”. “H/N” indicates heterozygous at this position. A phylogenetic tree was modified based on previous studies [5]

We aligned the amino acid sequences of SWS1, LWS, and RH1 from 13 species (Additional file 1: Fig. S1, Additional file 2: Table S1), and searched for amino acid replacements that affect the tuning of the absorption spectra of opsin pigments. In vertebrates, five positions, 164, 181, 261, 269, and 292 (positions corresponding to bovine RH1), in the middle to long wavelength sensitive opsins are known to affect absorption spectra of pigments [12, 17–20]. Among these spectral tuning positions, one amino acid replacement was found at position 292 in RH1 sequences and four replacements were found at positions 164, 181, 269, and 292 in LWS sequences (Fig. 1). In SWS1, two replacements (82S and 82Y) at position 82 (corresponding to position 85 in bovine RH1) was found in each of one Hydrophiini species (Additional file 1: Fig. S1c). Replacements at this position were reported to affect tuning of absorption spectra in SWS1 [53, 54]. Both replacements (F82S and F82Y) are estimated to cause long wavelength shifts of SWS1 pigments, and a polymorphism (F82 and Y82) at this position has persisted across Hydrophiini species [38]. Hereafter, we focused on *RH1* and *LWS* with replacements specific to lineages.

Next, we predicted the ancestral sequences of *RH1* and *LWS* and estimated the timing of amino acid replacements at spectral tuning positions. Serine at position 292 in RH1 was replaced by alanine (A) (represented as S292A) in the common ancestor of Hydrophiini and *Notechis* lineage (Fig. 1). Amino acid replacements, S164A, T269A, and A292S in LWS were found in Laticaudini species and in a pelagic Hydrophiini species, *Hydrophis platurus.* The replacements of these positions are shown on the branches in Fig. 1. The replacements, H181N and A292P, were found in *H. platurus* and two Laticaudini species, but the effects of these mutations on absorption spectra have not been reported previously.

To detect the effect of natural selection on opsin genes, we performed maximum likelihood approaches using the Codeml program. The results of a branch-site model test (foreground branches: *H. platurus* or Laticaudini and *H. platurus*; background branches: all other branches) suggested that the positions 179, 284, 307 in LWS (corresponding to positions 164, 269, and 292 in bovine RH1) have evolved under positive selection pressure (Additional file 3: Table S2).

Maximum likelihood trees for *SWS1*, *LWS*, and *RH1* genes were constructed. The sequences from each of Hydrophiini and Laticaudini formed a monophyletic clade in all trees, respectively (Additional file 4: Fig. S2).

To confirm the expression of opsin genes in a sea snake (*Emydocephalus ijimae*) and a terrestrial relative (*Sinomicrurus japonicus boettgeri*), we examined the expression of *RH1*, *SWS1*, and *LWS* genes by means of RNAseq analysis (Additional file 5: Fig. S3). Although we could not compare the expression difference due to one individual only from each species, the expression patterns of these three opsin genes from these species were similar to each other. Combining with no premature stop codon in these three genes (Additional file 1: Fig. S1), the expression patterns indicate that all three opsin genes are intact genes in sea snakes.

### Chromophore usage and functional diversification of RH1 and LWS pigments

Before in vitro reconstitution of visual pigments, it is necessary to know which type of chromophore is used in the eyes of the species under investigation. Comparing with the standards for A1- and A2-derived retinal (all-*trans*), the results of HPLC showed that the chromophore samples from all snake species, including Colubridae and Elapidae, contained A1-all-*trans* retinal (Additional file 6: Fig. S4). We used snake eyes without dark adaptation, therefore the isomer of A1-11-*cis* retinal (A1-all-*trans* retinal) was detected. According to this result, the chromophore usage of all snakes used in this study was estimated as A1-derived retinal (Table 1).

**Table 1:** Chromophore usage in snakes

Family	Subfamily	Genus	Species	Chromophore usage
Colubridae	Colubrinae	*Elaphe*	*climacophora*	A1-derived
		*Dinodon*	*rufozonatum walli*	A1-derived
		*Cyclophiops*	*herminae*	A1-derived
Elapidae	Laticaudinae	*Laticauda*	*semifasciata*	A1-derived
		*Laticauda*	*laticaudata*	A1-derived
	Hydrophiinae	*Hydrophis*	*melanocephalus*	A1-derived

**Fig. 2:**
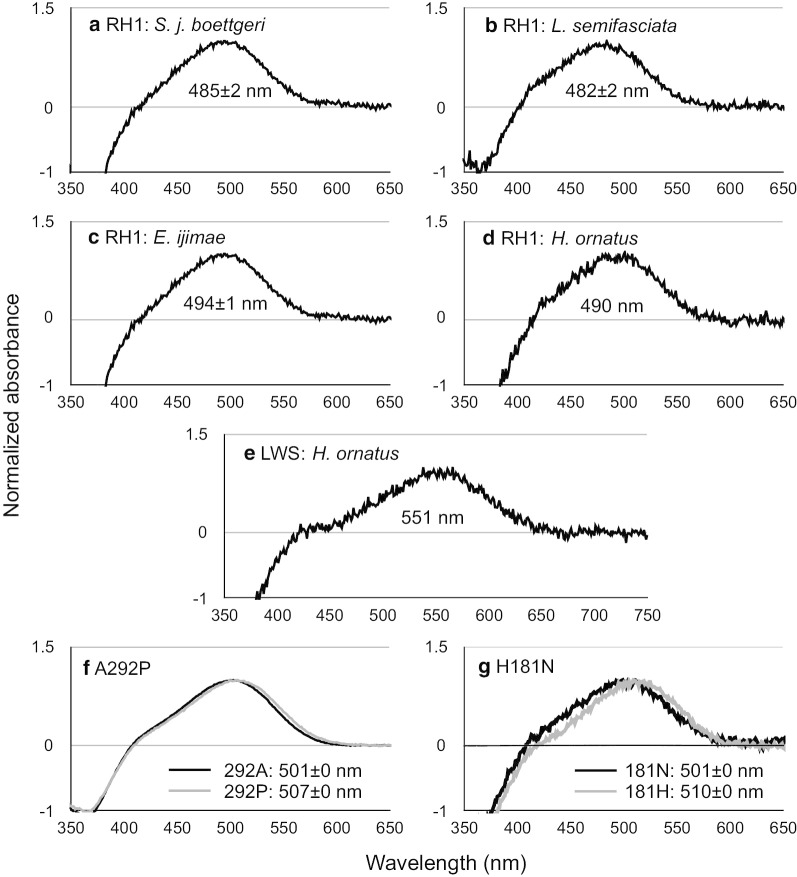
Absorption spectra of RH1 and LWS pigments evaluated by dark–light difference spectra. RH1 pigments were constructed from **a** *S. japonicus boettgeri*, **b** *L. semifasciata*, **c** *E. ijimae*, and **d** *H. ornatus*. LWS pigments were constructed from **e** *H. ornatus*. The absorption from mutants of RH1 pigments are shown in **f** A292 and 292P, and **g** 181H and 181N

**Fig. 3:**
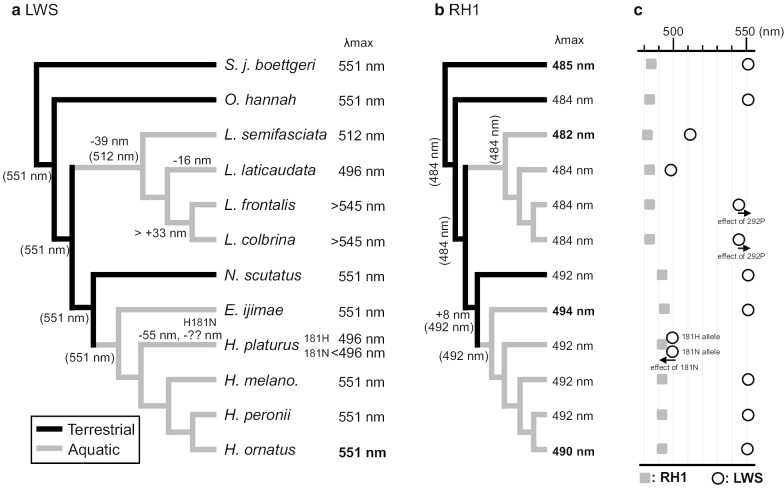
The λmax values for (**a**) LWS and (**b**) RH1. The values in bold and regular font are from measurements and prediction, respectively. The λmax values in ancestors are shown in parentheses on or under the branches. The values with plus and minus indicate red and blue shifts, respectively. Phylogenetic trees were modified based on previous studies [5]. Solid and gray blanches indicate terrestrial and aquatic lineages, respectively. **c** The λmax distributions are shown in gray boxes (RH1) and circles (LWS). The λmax values from *H. platurus* LWS are predicted from both the 181H and 181N alleles

To test whether *LWS* and *RH1* alleles were functionally different, we measured the difference in absorption spectra for each visual pigment that was reconstituted using A1-derived retinal. We measured reconstituted pigments from representative sequences with different predicted function of *RH1* (blue-shifted alleles with 292S compared with alleles with 292A), and several *LWS* sequences. However, except for from a *H. ornatus*, we did not obtain the absorption of pigments reconstituted from *LWS* sequences because of the instability of the in vitro reconstituted pigments or the low amount of viable pigment available in the present condition. The absorption spectra of the pigments were represented by the peak values (λmax), as shown in Fig. 2. The λmax values of RH1 pigments from the terrestrial snake, *S. japonicas boettgeri* (RH1: 292S), and Laticaudini sea snake, *L. semifasciata* (RH1: 292S), were 485 ± 2 nm and 482 ± 2 nm, respectively (Fig. 2a, b). On the other hand, that of Hydrophiini sea snakes *E. ijimae* (RH1: 292A) and *H. ornatus* (RH1: 292A) were 494 ± 1 nm and 490 nm, respectively (Fig. 2c, d). The predicted λmax values for *RH1* sequences without measurements were 484 nm and 492 nm (midpoint for each of two λmax of RH1 pigments with 292S and 292A) for sequences possessing 292S and 292A, respectively (Fig. 3).

In *LWS*, the λmax of pigments from the sequence of one Hydrophiini species, *H. ornatus*, was 551 nm (Fig. 2e). We used this λmax value for calibration and prediction of the λmax for other LWS pigments where reconstitution was unsuccessful. To predict the shift of the wavelength of the λmax values, we used the estimation of the spectral shift values for M/LWS pigments generated by amino acid changes reported [21]. The effects of H181N and A292P mutations on absorption spectra have however not been studied. To estimate the possible effect of amino acid changes (H181N and A292P), we introduced these amino acid residues in mouse *RH1* sequences by mutagenesis, and measured the absorption spectra. We used reconstituted mouse RH1 pigments due to its high stability. As shown in Fig. 2f, the λmax of the pigments from sequences with 292A and 292P were 501 ± 1 and 507 ± 1 nm, respectively, indicating that replacement of A292P induced a 6 nm shift toward red. The λmax of the pigments from sequences with 181H and 181 N were 510 ± 0 and 501 ± 0 nm, respectively, indicating that replacement of H181N induced a 9 nm shift toward blue. However, the shifts of λmax values by H181N and A292P were not directly applicable for the λmax value prediction of LWS pigments, because we used RH1 pigments for mutagenesis. In M/LWS, amino acid replacements at positions 181 and 292 have a synergistic effect on the shift of the λmax value [55]. Therefore, we predicted the possible effect of A292P and H181N replacements as red and blue shifts, respectively. At the positions 164, 269, and 292, the synergistic effects of the combinations of S164A/T269A and S164A/A292S were small (1 nm and 3 nm, respectively), or not known (S164A/T269A/A292S) [21]. Therefore, we did not take the synergistic effects of these combinations into account for the prediction of λmax values. From these results and the estimation in a previous study [21], we predicted that the amino acid changes S164A, T269A, and A292S generated shifts in the λmax values of -6, -16, and -33, respectively. The predicted λmax values for each opsin sequence and ancestral sequence are shown in Fig. 3. The λmax values for *L. frontalis* and *L. colbrina* LWS pigments were predicted by the effect of S164A and A292P.

### RH1 in amniotes

In total, 472 *RH1* sequences containing 236 mammalian, 102 avian, and 134 reptile (crocodiles, tortoises, lizards, and snakes) species were collected from the NCBI nucleotide database (Additional file 7: Table S3). Among them, all terrestrial species except snake species and the silvery mole-rat (*Heliophobius argenteocinereus*) had alanine at position 292 in RH1. Serine at this position was found in one fossorial silvery mole-rat and marine mammalian species such as cetaceans and the northern elephant seal (*Mirounga angustirostris*) (Additional file 7: Table S3). In contrast, 45 out of 113 terrestrial snake species possess a serine at this position (Additional file 7: Table S3). The serine at position 292 in RH1 from terrestrial species may be a specific feature in snakes.

### Prediction of the diving depth in sea snakes

**Fig. 4:**
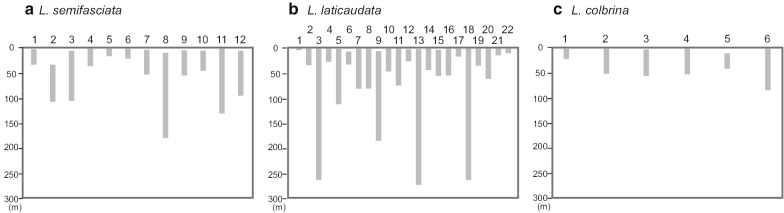
The depth distribution ranges of the prey fish species identified from the stomach contents of **a** *L. semifasciata*, **b** *L. laticaudata*, and **c** *L. colbrina*. The boxes indicate the water-depth distribution range of each species. Each lane indicates the species as follows: **a** 1 *Halichoeres trimaculatus,* 2 *Suezichthys soelae,* 3 *Acanthurus mata,* 4 *Ctenochaetus striatus*, 5 *Thalassoma amblycephalum,* 6 *Abudefduf sexfasciatus*, 7 *Parapercis clathrata*, 8 *Parapercis schauinslandii*, 9 *Caesio diagramma*, 10 *Chirrhitichthys aprinus*, 11 *Grammistes sexlineatus*, 12 *Centropyge heraldi*; **b** 1 *Cirrimaxilla Formosa*, 2 *Myrichthys colubrinus*, 3 *Myrichthys maculosus*, 4 *Scolecenchelys laticaudata*, 5 *Gymnothorax undulates*, 6 *G. margaritophorus*, 7 *Conger* cf. ‘cinereus’, 8 *C. cinereus*, 9 *G. albimarginatus*, 10 *G. chilospilus*, 11 *G. eurostus,* 12 *G. moluccensis*, 13 *G. nudivomer*, 14 *G. pindae*, 15 *G. reevesi*, 16 *G. reticulari*s, 17 *G. richardsonii*, 18 *Myrichtys maculosus*, 19 *Myrophis microchir*, 20 *Plotosus lineatus*, 21 *Strophidon sathete*, 22 *Uropterygius concolor*; **c** 1 *Echidna polyzona* 2 *Echidna nebulosi*, 3 *Gymnomuraena zebra*, 4 *G. javanicus*, 5 *Uropterygius nagoensis*, 6 *C. cinereus*

To predict the distribution of diving depth, we searched the distribution of prey species found in the stomach contents of sea snakes, *Laticauda semifasciata*, *L. laticaudata, L. colbrina, H. platurus, H. ornatus,* and *E. ijimae* [56–61]. The distributions of prey fish species were searched using FishBase (https://www.fishbase.se/search.php). The depth distribution of fish species from stomach contents provides direct evidence for the minimum diving depth of Laticaudini species (Fig. 4). The average depths of the midpoint of the distributions of the prey fish species were 36, 41, and 25 m for *L. semifasciata*, *L. laticaudata,* and *L. colbrina*, respectively. In Hydrophini species, one goby species, *Oplopomus oplopomus,* has been reported form a stomach content of *H. ornatus* [61]. The depth distribution of this goby species ranges from 1 to 20 m. Only eggs have been reported from the stomach contents of *E. ijimae*, and the eggs can be traced to bottom-dwelling inshore species of at least two families, Gobiidae and Blenniidae [59]. A pelagic species, *H. platurus*, preys swimmer type small surface-living epipelagic fishes [60].

## Discussion

### Visual adaptation of color opsins in sea snakes

Snakes are one of the most successful amniote groups to adapt to various environments. They inhabit a diversity of environments on all continents except for Antarctica. Among them, sea snakes have adapted to the marine environment. It has been found that snakes including fully-aquatic hydrophiins rely heavily on olfaction for recognizing surrounding environments [3, 62]. However, snakes also rely on vision, and sea snakes find mates and locate the substrates where their prey is hiding using visual cues [63, 64].

In this study, we focused on *LWS* and *RH1* and found amino acid variations at several spectral positions. As shown in Fig. 3, we summarized the results of measurements and prediction of the λmax values for LWS and RH1 pigments.

In the LWS, amino acids at three spectra tuning positions have been replaced in laticaudins (Fig. 1). Compared to their common ancestor, S164A and A292S replacements are present in this group (Fig. 1). These two replacements were predicted to shift the absorption 39 nm towards blue. Thus, the LWS pigments in the MRCA of laticaudins were blue shifted (Fig. 3, λmax: 512 nm). Subsequently, a replacement T269A has caused a 16 nm shift toward blue (λmax: 496 nm) in *L. laticaudata* (Fig. 3). The λmax values of LWS and RH1 pigments are close to each other in this species (Fig. 3c).

Water scatters and absorbs blue and red light, respectively. As a consequence, blue light (centered around 475 nm) remains in the deep water environment [9]. Therefore, it is reasonable to assume that *L. semifasciata* and *L. laticaudata* with blue shifted LWS pigments dive deeper and have adapted to the ambient blue light environment. However, little is known about the ecology of *Laticauda* species, especially diving depth. In order to investigate their diving ecology, we examined the stomach contents of three laticaudins. The average distribution depths of the prey fish species of *L. semifasciata* and *L. laticaudata* were deeper than that of *L. colbrina*. Moreover, the maximum depth distribution of the prey fish for *L. semifasciata* and *L. laticaudata* were over 100 m (five out of twelve species) and 200 m (three out of 22 species), respectively (Fig. 4a, b). The average distribution depths of the prey fish species may reflect difference in hunting depths between laticaudins. Therefore, the blue shifted LWS pigments in *L. semifasciata* and *L. laticaudata* may have adapted to the deeper water light environment.

In addition to the blue shifts, a red shift was also predicted in the LWS pigments in the MRCA of *L. colbrina* and *L. frontalis* with a S292P replacement. According to the effect of a replacement in RH1 pigments, the replacement A292P in LWS pigments was predicted to cause a possible shift toward red. Therefore, the λmax value of LWS with 164S and 292P may be larger than those with 164S and 292A (545 nm). As discussed above, the average distribution depths of the prey fish species of *L. colbrina* were shallower than that of *L. semifasciata* and *L. laticaudata* (Fig. 4c), indicating that the red shift may be an adaptation to the shallow water light environment.

In all hydrophiins except for *H. platurus*, the amino acids of LWS at the spectra tuning positions are identical to terrestrial snakes (Fig. 1). The stomach contents of *H. ornatu*s and *E. ijimae* are inshore fish or inshore fish eggs, indicating that these two species hunt in shallow water. The light environment in shallow water is composed of a broad spectrum and is similar therefore to the terrestrial light environment. On the other hand, *H. melanocephalus* has been observed at 44 m [65], and mainly preys on eel-shaped fish dwelling within burrows along the bottom [60]. *H. peronii* were collected from deep sand bottom [66]. This species mainly preys goby-shape fish [60] and captures its prey in the goby borrows [66]. Although *H. melanocephalus* and *H. peronii* have been reported from relatively deep water, the predicted absorption of LWS pigments are the same as that of the terrestrial snakes. The red-shifted terrestrial type *LWS* in these species could be explained by their hunting environment, which is likely to exhibit low transparency because of the sandy/muddy subtrate in the hunting habitat.

Amino acids at three spectral tuning positions (164, 269, and 292) have been replaced in the LWS of *H. platurus* (Fig. 1). These three replacements were predicted to cause a 55 nm shift toward blue (λmax: 496 nm) in the LWS pigments of this species. In addition, one of the alleles from this individual (181 N allele) possesses a replacement at another spectral tuning position (181 N). According to the effect of a replacement in RH1 pigments, a replacement H181N may cause a possible blue shift in the LWS pigments. In sea snakes, only *H. platurus* is a pelagic species [1, 67]. *H. platurus* preys small epipelagic fishes, therefore the LWS pigments in *H. platurus* may have adapted to open ocean environments dominated by blue light.

Three (positions, 164, 269, and 292) out of four replacements at spectra tuning positions in *H. platurus* were also replaced by the same amino acid residues observed in Laticaudini species, indicating parallel amino acid replacements in different lineages. Parallel amino acid replacement can be viewed as evidence of natural selection. Indeed, natural selection has acted on these three positions (Additional file 3: Table S2), supporting adaptation to environments dominated by blue light.

### The evolutionary pattern of RH1 in snakes differs from that of other vertebrates

In RH1, the amino acid at spectra tuning position 292 is serine in Laticaudini species, while those in Hydrophiini species, it is alanine. In general, *RH1* sequences with 292A are observed in vertebrates living in shallow water [10] as well as terrestrial amniotes except for snakes (Additional file 7: Table S3). On the other hand, 292S has been reported from *RH1* of deep-diving marine mammals [42–44] as well as fish living in the deep sea and high transparency lakes [10, 68], suggesting deep water adaptation. According to this pattern, the 292S in deep water Laticaudini and 292A in Hydrophiini species with different λmax values are presumed to be the result of adaptation to deep and shallow water environments, respectively. However, S292A has occurred in the MRCA of Hydrophiini and the terrestrial tiger snake (*Notechis scutatus*). In addition, the *RH1* of terrestrial ancestors possessed 292S (Fig. 1), suggesting that the function of snake RH1 pigments may differ from those of other terrestrial amniote species. In snakes, many terrestrial species possess *RH1* with 292S (Additional file 7: Table S3), and 292A and 292S have been acquired multiple times during snake evolution [37]. What is the difference in the role of RH1 pigments between snakes and other terrestrial amniotes? RH1 is expressed in rod cells in most amniotes [10]. However, rod cells have been “transmutated” and function as cone-like cells in several snake lineages [32]. This cone-like rod cell is also reported from Hydrophiini [33, 34], and RH1 is localized in cone-like cells in Colubridae species [30]. Although the adaptive role of *RH1* with 292A and 292S in snakes remains unclear, the RH1 pigments that differ for these two amino acid residues may have different spectral sensitivity in cone-like cells, possibly in color vision (conditional trichromacy).

### Comparison of evolution of opsins between sea snakes and other aquatic amniotes

**Fig. 5:**
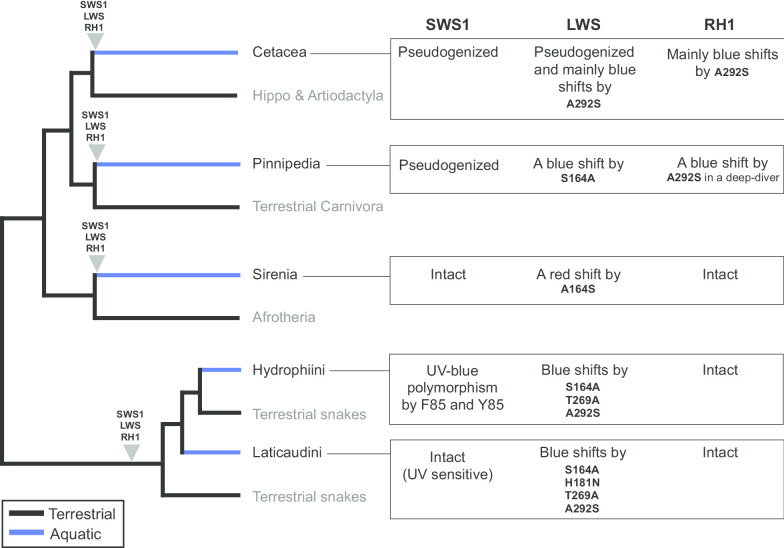
Comparison of opsin evolution between aquatic amniote groups

Each of the MRCAs of marine mammals and sea snakes had three visual opsins; SWS1, LWS, and RH1, respectively [10], indicating the common ancestors of each of the groups initially invaded an aquatic environment with the same opsin gene repertoires (summarized in Fig. 5). We compared the evolution of opsins in marine mammals (cetacea, sirenia, and pinnipedia) and sea snakes (summarized in Fig. 5).

Among marine amniote groups, the time scales after adaptation to an aquatic environment are different. Each of the MRCAs of cetacea, sirenia, and pinnipedia adapted to the aquatic environment 29.4, 31.4 and 21.3 Million years ago (MYA), respectively [69]. On the other hand, the aquatic adaptation of the MCRA of hydrophiins have been estimated to be ~ 6 MYA [4, 70], and that of laticaudins is estimated to be younger than the split of Hydrophiini and Laticaudini (12–20 MYA) [2, 4, 7]. Thus, the aquatic adaptation of sea snakes are younger than those of marine mammals.

The main difference between these marine amniote groups was pseudogenization of opsin genes. In sea snakes and sirenians, all three opsin genes (*SWS1*, *LWS*, and *RH1*) are intact (Fig. 5). Species in sirenia have adapted to shallow water and have maintained three opsins as intact genes [71]. In contrast, *SWS1* was independently pseudogenized in the ancestral lineages of toothed and baleen whales, and five independent pseudogenization events in *LWS* occurred in deep-diving cetacean lineages, leading to rod monochromatic vision in several cetacean species [51]. Moreover, the pseudogenization of the *SWS1* gene has also occurred in the pinniped lineage [45, 46, 48, 52], resulting in color blindness. Rod monochromatic cetacean species are known to dive to depths of at least 100 m where the light environment is blue dim light [51]. Pseudogenization of *SWS1* and *LWS* genes could be the result of natural selection favoring an all-rod retina in blue dim light [51]. Meredith et al. discussed one possibility that cone opsins have a higher rate of dark noise (output signals without light inputs) than RH1, and may interfere with rod sensitivity under scotopic conditions [51]. Based on this scenario, SWS1 and LWS in sea snakes may not interfere with rod sensitivity in dim light, because the rod cells have transmuted into cone-like cells [33, 34]. This might be one possibility for the difference in pseudogenization events between deep diving cetaceans and sea snake species.

As mentioned above, transmutation from rod cells to cone-like cells has been reported from Hydrophiini species including *H. platurus,* and this has resulted in the formation of all cone retinas [33, 34]. Therefore, the RH1 pigments in Hydrophiini species (perhaps also in Laticaudini) may contribute to color vision, a form of conditional trichromacy. In this case, sea snakes have three color opsins; short (*SWS1*), middle (*RH1*), and long (*LWS*) wavelength sensitive, while cetaceans have one *(LWS*) or no color opsins. In sea snakes, three color opsins could increase spectral sensitivity and chromatic discrimination with the potential for color vision. The blue shifts of LWS pigments with different λmax values may contribute to this conditional trichromacy. On the other hand, LWS pigments in cetacean species may not contribute to chromatic discrimination, resulting in blue shifts by one amino acid replacement, A292S [42, 43]. According to the discussion for different evolution of amino acids at position 292 in *RH1*, differences in pseudogenization, and blue shifts of LWS pigments, we hypothesized that the types of retina, cone and rod or all-cone, may have affected the pseudogenization of opsin genes and the spectral shift of opsin pigments during adaptation to an aquatic environment. However, the evidence supporting this hypothesis is still insufficient. Further analyses of retina and opsin pigments in sea snake species should reveal the answer to this hypothesis.

## Methods

### Samples

Samples used in this study were collected through fieldwork in the Ryukyu area in Japan. Collected species are as follows: *S. japonicus boettgeri* (Nago, Okinawa, specimen voucher: KUZR072621), *L. semifasciata* (Uganzaki, Ishigaki island), *L. laticaudata* (Uganzaki, Ishigaki island), *L. colubrina* (Noharazaki, Ishigaki island, specimen voucher: KUZR66612), *E. ijimae* (Maeda, Okinawa, specimen voucher: KUZR072604), *H. melanocephalus* (Okinawa, specimen voucher: KUZR072403) and *H. ornatus* (Motobu, Okinawa, specimen voucher: KUZR72684). The minimum number of specimens (one individual per species) were caught and euthanased by anaesthetic injection (somnopentyl, Kyoritsu Seiyaku Corporation, Tokyo, Japan). The snake eyes and muscle tissues were surgically removed and stored in RNAlater or kept in the freezer (-20 °C). Genomic DNA of *L. frontalis* extracted by Kishida et al. [72] and that of *H. platurus* extracted by Kishida and Hikida [73] were also used in this study (listed in Additional file 2: Table S1).

### Determination of opsin genes sequences

Total RNAs were extracted from eyes of *S. japonicus boettgeri*, *L. semifasciata, L. laticaudata, E. ijimae, H. melanocephalus* and *H. ornatus* using TRIzol RNA Isolation Reagent (Thermo Fisher Scientific, Waltham, MA, USA) according to the manufacturer’s instructions. Extracted total RNAs were purified using RNeasy mini kit (Qiagen, Hilden, Germany) following the manufacturer’s protocol. First-strand cDNA was reverse-transcribed from 500 ng of the eye total RNA using a PrimeScript RT Reagent Kit with gDNA Eraser (Takara, Shiga, Japan). Genomic DNAs were extracted from tissues of *L. colbrina* using the DNeasy Blood & Tissue Kit (Qiagen). The opsin sequences of *O. hannah* (GCA_000516915.1), *L. laticaudata* (GCA_004320025.1), and *H. melanocephalus* (GCA_004320005.1) were isolated by BLASTN [74] using the *Anolis carolinensis SWS1* (XM_003229162), *LWS* (XM_008103916), and *RH1* (NM_001291387) sequences as queries. Specific primers to amplify exonic regions of opsin genes were designed on the conserved regions among three species, *Ophiophagus hannah* (GCA_000516915.1), *L. laticaudata* (GCA_004320025.1), and *H. melanocephalus* (GCA_004320005.1). The primer sequences are listed in Additional file 8: Table S4. Coding regions of opsins were amplified by PCR using cDNAs as templates with the following conditions: a denaturation step for 3 min at 94 °C followed by 35 cycles of denaturation for 1 min at 94 °C, annealing for 1 min at 55 °C, and extension for 2 min at 72 °C. Each of the exons of opsins were amplified by PCR using gDNAs as templates with the same condition for cDNA templates. PCR products were purified and the sequences were determined using the Applied Biosystems Automated 3130xl Sequencer (Applied Biosystems, Waltham, MA, USA).

The opsin sequences of *Notechis scutatus*, *Alizona elegans*, and *H. peroneii* (Additional file 2: Table S1) were collected from NCBI nucleotide database (http://blast.ncbi.nlm.nih.gov).

### RNA library construction, sequencing, and expression analysis

RNA libraries were constructed using the NEBNext Poly(A) mRNA Magnetic Isolation Module and the NEBNext Ultra RNA Library Prep Kit for Illumina (New England Bio Labs, Ipswich, MA) following the manufacturer’s instructions. Short cDNA sequences (paired-end 125 bp) were determined from the libraries using the Illumina Hiseq2500 platform (RNA-seq). RNA-seq reads from each of one individual of *S. japonicus boettgeri* and *E. ijimae* (9.3 and 10.1 Gbp, respectively) were mapped to the sequences of *SWS1*, *LWS*, and *RH1* from each species. Reads showing similarity (90%) with 90% read lengths were mapped to opsin sequences and expression levels were calculated using CLC Genomics Workbench ver. 11 (https://www.qiagenbioinformatics.com/). TPM (Transcript Per Million) was used for normalized expression values.

### Evolutionary analyses

All opsin sequences obtained were aligned with GENETYX ver. 19.0 software (Genetyx Corporation, Tokyo, Japan). Phylogenetic analyses were conducted using Maximum Likelihood approaches. The evolutionary history was inferred by using the Maximum Likelihood method based on the Tamura-Nei model [75]. The percentage of trees in which the associated taxa clustered together is shown next to the branches. The tree is drawn to scale, with branch lengths measured in the number of substitutions per site. All positions containing gaps and missing data were eliminated. Evolutionary analyses mentioned above were conducted using MEGA ver. 7.0 software [76]. The sequence from *A. elegans* was used as an out group.

The effect of natural selection acting on the opsin genes was assessed in Elapidae snakes including sea snakes by maximum likelihood approaches using the Codeml program in the PAML 4.8 package [77]. This program was used to estimate the ratio (ω) of the non-synonymous substitution (dN) to the synonymous substitution (dS) rate across study species and within the individual genes. Selection tests were performed with branch models, site models, and branch-site models. We defined four ecological states for each species as follows: Laticaudini is ‘semi-aquatic’, Hydrophini is ‘full-aquatic’, *H. platurus* is ‘plagic’, and terrestrial species is ‘terrestrial’. Species trees were referenced according to Zaher et al. [5].

### Chromophore determination

Without dark adaptation, the snake eyes were surgically removed from the head and kept in the freezer (− 20 °C) under dark conditions until the analysis for their chromophore composition. The eyes were returned to a temperature slightly higher than the ice temperature, and the anterior part including the lens was removed and the vitreous was drained. The posterior part, the eyecup was transferred into a glass homogenizer on ice and was homogenized in 200 µL phosphate-buffered physiologic saline. The chromophores of the visual pigments were converted to their oxime forms using the method of Groenendijk et al. [78], as modified by Suzuki et al. [79]. Briefly, homogenates were treated with 200 µL of 1 M hydroxylamine (pH 6.0) and 0.5 mL methanol to obtain isomers of the pigment chromophores as oximes. The oximes were extracted by 0.5 mL of dichloromethane and 1 mL of *n*-hexane and the solvent was removed by a fine flow of nitrogen gas.

The oximes of chromophores were analyzed using HPLC. The elution solvent was 7% diethyl-ether in hexane containing 0.075% ethanol, and the flow rate was kept constant at 1.5 mL min by a model 880-PU pump (JASCO Co., Ltd.). The dried extracts were dissolved by 50 µL of the elution solvent and were injected into a Waters-type A-012 (SIL) packed column (6 × 150; Yamamura Chemical Laboratories Co., Ltd., Kyoto, Japan). Absorbance was monitored at 360 nm with a model 870-UVi spectrophotometer (JASCO Co., Ltd.), and automatically recorded and calibrated (CDS-Lite Ver.5.0 LA soft Co. Ltd.). The extracts were compared with A1- and A2-derived retinal (all-*trans*) standards obtained Sigma-Aldrich Co. LLC and TRC Inc.

### Reconstruction and measurement of visual pigment absorption spectra

Production, reconstruction, purification, and measurement of the visual pigments were performed as described previously [80], with minor modifications. Briefly, the opsin sequences were amplified by PCR using cDNA as a template with a pair of specific PCR primers (Additional file 8: Table S4) designed to produce a fusion protein with a FLAG-tag (Sigma-Aldrich; St. Louis, Missouri, United States) at the C terminus. The amplified DNA fragments were digested with restriction enzymes and subcloned into the expression vector pFLAG-CMV-5a (Sigma-Aldrich). The constructs of mouse RH1 with asparagine at the position 181 (181 N), histidine at 181 (181H), and proline at 292 (292P) were developed from the mouse RH1 construct by PCR-based mutagenesis [81]. PCR primers for mutagenesis are listed in Additional file 8: Table S4. The visual pigments were reconstituted with A1-derived retinal. Absorption spectra of the pigment solutions in the presence of hydroxyl-amine (< 100 mM) before and after photobleaching were recorded using a spectrophotometer (UV-1800; Shimadzu; Kyoto, Japan), with 5 measurements before and after photobleaching by LED light (505 nm). The mean peak spectral values (maximum absorption spectra: λmax) and standard errors were determined from multiple (two or three times) preparations and measurements for each pigment. After reconstitution of the pigments, all procedures were performed under dim red (> 680 nm) or infrared light (> 900 nm) using a digital video camera recorder (DCR-TRV8; Sony) in “night shot” mode or in complete darkness.

### RH1 sequences of Amniota species in database

Sequences of the *RH1* gene in amniota were collected by BLASTN [74] from the NCBI nucleotide database of mammalia (taxid:40,674), aves (taxid:8782), and sauropsida (taxid:8457) using *RH1* sequences of *Bos taurus* (NM 001014890.2), *Gallus gallus* (NM 001030606.1), and *O. hannah* (isolated from GCA_000516915.1) as queries, respectively.

## Supplementary information

**Additional file 1: Figure S1.** Alignments of (a) LWS, (b) RH1, and (c) SWS1 amino acid sequences.

**Additional file 2: Table S1.** Species and opsin sequences used in this study.

**Additional file 3: Table S2.** Amino acid sites inferred to be under positive selection, identified under branch-site models for the three visual opsin genes.

**Additional file 4: Figure S2.** Maximum Likelihood trees for (a) *SWS1*, (b), *LWS*, and (c) *RH1. *Bootstrap probability for each clade was obtained by 1,000 replicates and is shown next to each node. The scale bar represents 0.01 (SWS1) and 0.005 (LWS and RH1) substitutions per site.

**Additional file 5: Figure S3.** Relative expression of three opsins in (a) *S. japonicus boettgeri *and (b) *E. ijimae*.

**Additional file 6: Figure S4.** Results of HPLC analysis.

**Additional file 7: Table S3.** RH1 with 292A and 292S in amniota species.

**Additional file 8: Table S4.** Primer sequences.

## Data Availability

The nucleotide sequences were deposited in GenBank under accession numbers LC543589–LC543615; DRX218721–DRX218722.

## Abbreviations

λmax: Peak absorbance
SWS1: Short wavelength sensitive opsin 1
SWS2: Short wavelength sensitive opsin 2
LWS: Long wavelength sensitive opsin
RH1: Rhodopsin 1
RH2: Rhodopsin 2
Ma: Million years
MYA: Million years ago
MRCA: The most recent common ancestor
HPLC: High performance liquid chromatography
cDNA: Complementary DNA
PCR: Polymerase chain reaction
RNA-seq: Short cDNA sequencing
TPM: Transcript per million
