# Supplementary material for: Visual adaptation of opsin genes to the aquatic environment in sea snakes
Source: BMC Evol Biol. 2020 Nov 26;20:158. doi: 10.1186/s12862-020-01725-1 (PMC7690139; doi:10.1186/s12862-020-01725-1)
Supplement: Supplementary file 2 — Additional file 2: Table S1. Species and opsin sequences used in this study. [file 12862_2020_1725_MOESM2_ESM.pdf]

Table S1 Species and opsin sequences used in this study

| Superfamily | Family | Subfamily | Tribe | Genus | Species | Number of individual | *SWS1* | *LWS* | *RH1* | Locality |
|---|---|---|---|---|---|---|---|---|---|---|
| Colubroidea | Colubridae | Colubrinae | - | *Arizona* | *elegans* | - | KU323997 | KU323986 | KU324006 | - |
| | Elapidae | Elapinae | - | *Sinomicrurus* | *japonicus boettgeri* | 1 | LC543607 | LC543589 | LC543598 | Nago, Okinawa |
| | | | - | *Ophiophagus* | *hannah* | - | Isolated from genome sequence | | | - |
| | | Laticaudinae | Laticaudini | *Laticauda* | *semifasciata* | 1 | LC543608 | LC543590 | LC543599 | Uganzaki, Ishigaki island |
| | | | | *Laticauda* | *laticaudata* | 1 | LC543609 | LC543591 | LC543600 | Uganzaki, Ishigaki island |
| | | | | *Laticauda* | *frontalis* | - | LC543610 | LC543592 | LC543601 | From Kishida et al. (2013) |
| | | | | *Laticauda* | *colubrina* | 1 | LC543611 | LC543593 | LC543602 | Noharazaki, Ishigaki island |
| | | Acanthophiinae | - | *Notechis* | *scutatus* | - | KU323999 | KU323989 | KU324000 | - |
| | | Hydrophiinae | Hydrophiini | *Emydocephalus* | *ijimae* | 1 | LC543612 | LC543594 | LC543603 | Maeda, Okinawa |
| | | | | *Hydrophis* | *platurus* | - | LC543613 | LC543595 | LC543604 | From Kishida and Hikida (2010) |
| | | | | *Hydrophis* | *melanocephalus* | 1 | LC543614 | LC543596 | LC543605 | Okinawa |
| | | | | *Hydrophis* | *peronii* | - | KU323991 | KU323990 | KU324001 | - |
| | | | | *Hydrophis* | *ornatus* | 1 | LC543615 | LC543597 | LC543606 | Motobu, Okinawa |
