# Supplementary material for: Visual adaptation of opsin genes to the aquatic environment in sea snakes
Source: BMC Evol Biol. 2020 Nov 26;20:158. doi: 10.1186/s12862-020-01725-1 (PMC7690139; doi:10.1186/s12862-020-01725-1)
Supplement: Supplementary file 3 — Additional file 3: Table S2. Amino acid sites inferred to be under positive selection, identified under branch-site models for the three visual opsin genes. [file 12862_2020_1725_MOESM3_ESM.pdf]

Table S2. Amino acid sites inferred to be under positive selection, identified under branch-site models for the three visual opsin genes.

Gene	Foreground branch	Sites under positive selection	2Δ(ln *L*)	P
SWS1	Laticaudini (amphibious)	11 - 52 - 107 - 278*	4.945	0.026*
	Pelagic Hydrophiini (*H. platurus*)	328	0	1
	Laticaudini + Pelagic Hydrophiini (*H. platurus*)	52 - 222 - 278	2.599	0.107
	Hydrophiini (fully-aquatic + pelagic)	5 - 10 - 82 - 104 - 161 - 258	3.465	0.063
	Laticaudini+ Hydrophiini (fully-aquatic + pelagic)	5 - 10 - 82 - 104 - 258 - 276 - 278	2.480	0.115
RH1	Laticaudini (amphibious)	None	-	-
	Pelagic Hydrophiini (*H. platurus*)	None	-	-
	Laticaudini + Pelagic Hydrophiini (*H. platurus*)	None	-	-
	Hydrophiini (fully-aquatic + pelagic)	19 - 104 - 173 - 189 - 198 - 225 - 239 - 304	1.138	0.286
	Laticaudini+ Hydrophiini (fully-aquatic + pelagic)	189	0.124	0.725
LWS	Laticaudini (amphibious)	None	0.241	0.624
	Pelagic Hydrophiini (*H. platurus*)	28* - 177* - **179*** - 181* - **284*** - 285* - **307***	0.842	0.359
	Laticaudini + Pelagic Hydrophiini (*H. platurus*)	24 - 25 - 28* - 40 - 67 - 103 - 119 - 124 - 177 - **179*** - 181 - 228 - 233 - **284*** - 285 - 297 - 305 - **307****	17.345	3.117E-05**
	Hydrophiini (fully-aquatic + pelagic)	180	0	1
	Laticaudini+ Hydrophiini (fully-aquatic + pelagic)	28 - 114 - 124 - 179 - 180 - 181 - 284 - **307***	9.360	0.002*

Positions in bold are those known to be associated with spectral tuning. * 0.05, ** 0.01
