# Supplementary material for: Visual adaptation of opsin genes to the aquatic environment in sea snakes
Source: BMC Evol Biol. 2020 Nov 26;20:158. doi: 10.1186/s12862-020-01725-1 (PMC7690139; doi:10.1186/s12862-020-01725-1)
Supplement: Supplementary file 4 — Additional file 4: Figure S2. Maximum Likelihood trees for (a) SWS1, (b), LWS, and (c) RH1. Bootstrap probability for each clade was obtained by 1,000 replicates and is shown next to each node. The scale bar represents 0.01 (SWS1) and 0.005 (LWS and RH1) substitutions per site. [file 12862_2020_1725_MOESM4_ESM.pdf]

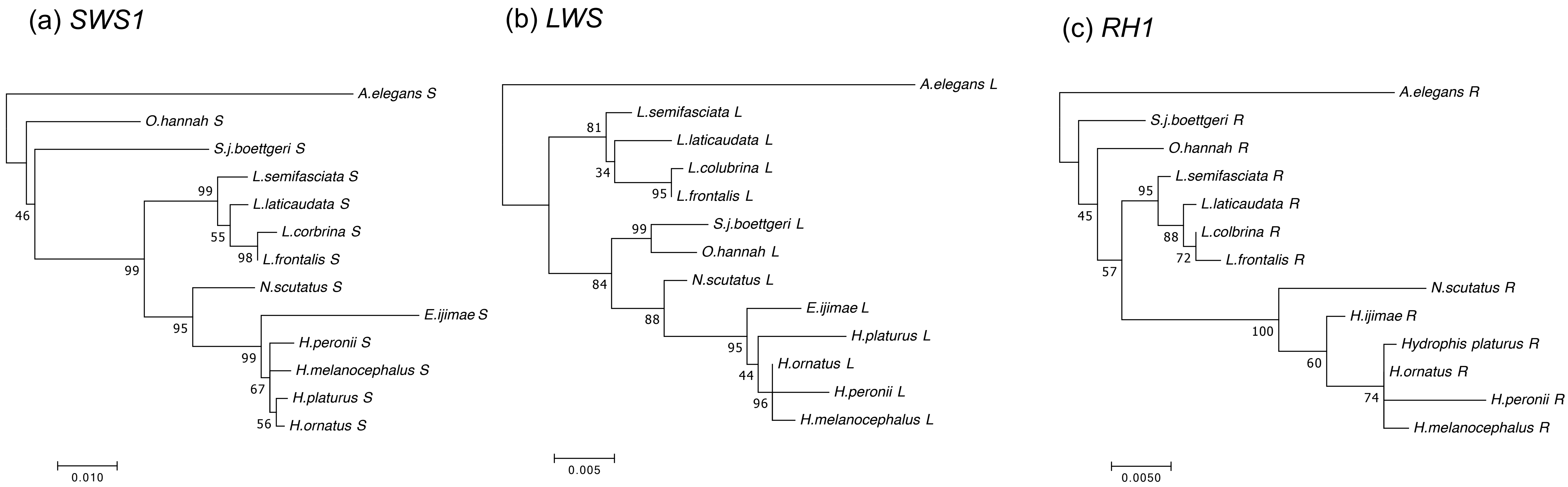

Fig. S2 Maximum Likelihood trees for (a) *SWS1*, (b), *LWS*, and (c) *RH1*
Bootstrap probability for each clade was obtained by 1,000 replicates and is shown next to each node. The scale bar represents 0.01 (SWS1) and 0.005 (LWS and RH1) substitutions per site.
