# Supplementary material for: Visual adaptation of opsin genes to the aquatic environment in sea snakes
Source: BMC Evol Biol. 2020 Nov 26;20:158. doi: 10.1186/s12862-020-01725-1 (PMC7690139; doi:10.1186/s12862-020-01725-1)
Supplement: Supplementary file 5 — Additional file 5: Figure S3. Relative expression of three opsins in (a) S. japonicus boettgeri and (b) E. ijimae. [file 12862_2020_1725_MOESM5_ESM.pdf]

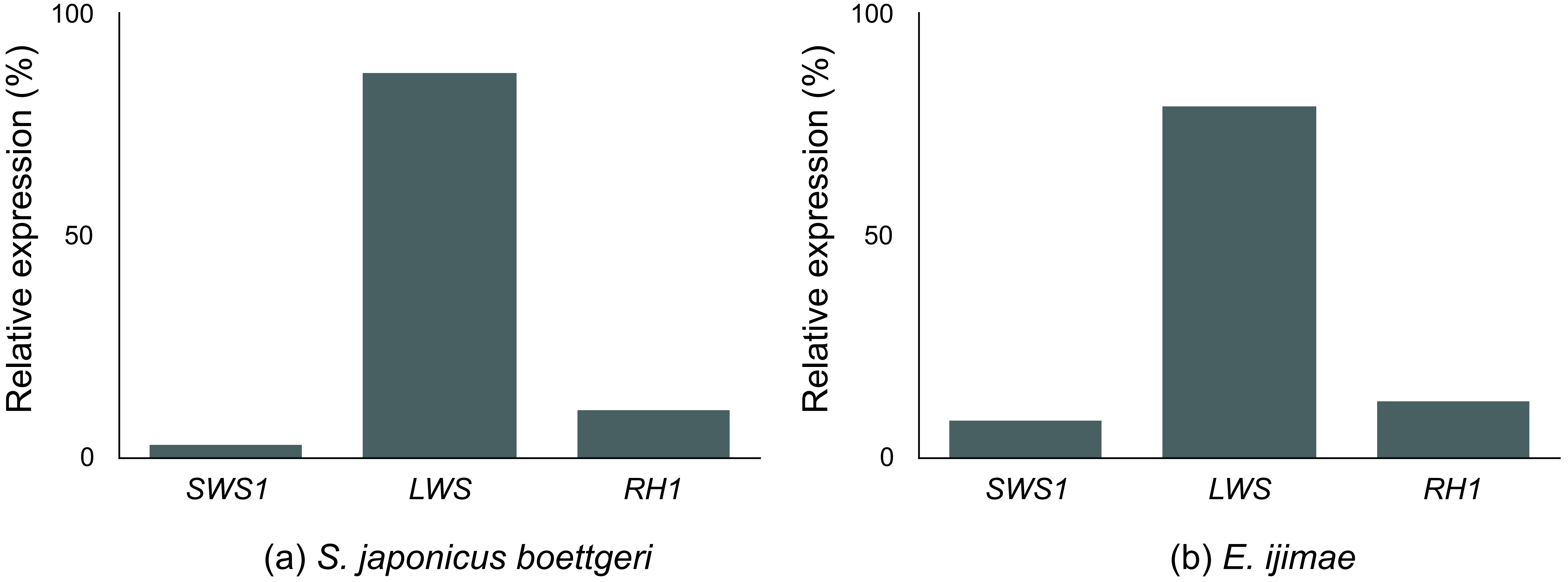

Fig. S3 Relative expression of three opsins in (a) *S. japonicus boettgeri* and (b) *E. ijimae*.
