# Supplementary figures and images for: Visual adaptation of opsin genes to the aquatic environment in sea snakes
Source: BMC Evol Biol. 2020 Nov 26;20:158. doi: 10.1186/s12862-020-01725-1 (PMC7690139; doi:10.1186/s12862-020-01725-1)

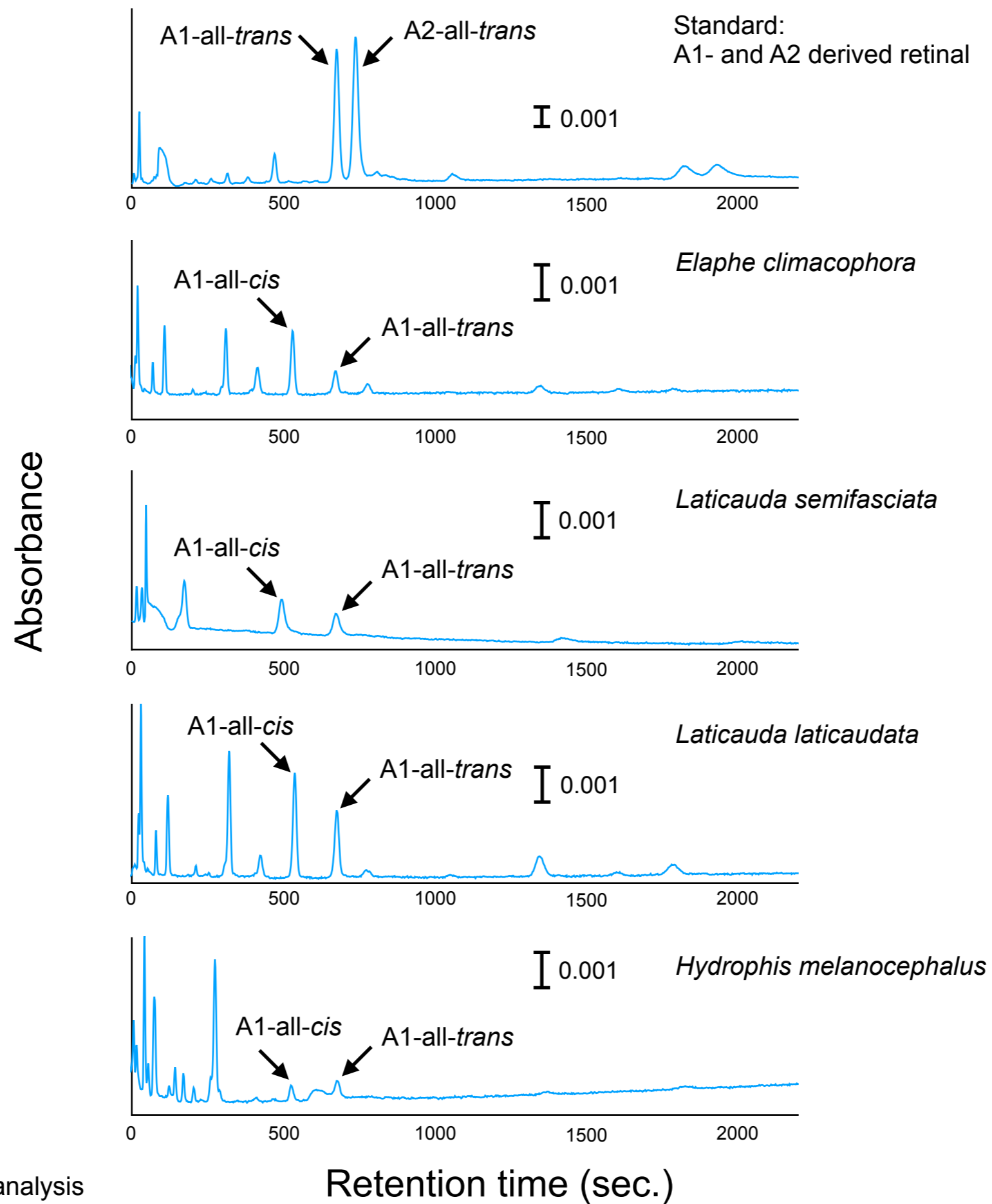

Fig. S4 Results of HPLC analysis

Supplement: Supplementary file 6 — Additional file 6: Figure S4. Results of HPLC analysis. [file 12862_2020_1725_MOESM6_ESM.pdf]
