# Supplementary material for: Visual adaptation of opsin genes to the aquatic environment in sea snakes
Source: BMC Evol Biol. 2020 Nov 26;20:158. doi: 10.1186/s12862-020-01725-1 (PMC7690139; doi:10.1186/s12862-020-01725-1)
Supplement: Supplementary file 7 — Additional file 7: Table S3. RH1 with 292A and 292S in amniota species. [file 12862_2020_1725_MOESM7_ESM.pdf]

Table S3. RH1 with 292A and 292S in amniota species

Taxonomy			Species	accession numbers	RH1 292
Sauropsida	Archosauria	Crocodylia	*Alligator mississippiensis*	NM_001287282.1	A
			Alligator sinensis	XM_006039400.2	A
			Crocodylus porosus	XM_019536553.1	A
			Gavialis gangeticus	XM_019504656.1	A
		Avian	*Aquila chrysaetos chrysaetos*	XM_029996218.1	A
			Chaetura pelagica	XM_010003384.1	A
			Calypte anna	XM_008501365.1	A
			Buceros rhinoceros silvestris	XM_010145186.1	A
			Antrostomus carolinensis	XM_010172233.1	A
			Cariama cristata	XM_009709088.1	A
			Charadrius vociferus	XM_009882892.1	A
			Calidris pugnax	XM_014946656.1	A
			Colius striatus	XM_010196121.1	A
			Columba livia	AH007730.2	A
			Streptopelia turtur	LR594561.1	A
			Leptosomus discolor	XM_009949111.1	A
			Cuculus canorus	XM_009565824.1	A
			Haliaeetus albicilla	XM_009914629.1	A
			Haliaeetus leucocephalus	XM_010570157.1	A
			Falco cherrug	XM_005443603.3	A
			Falco peregrinus	XM_005240222.3	A
			Anas platyrhynchos	AF021240.1	A
			Anser cygnoides domesticus	XM_013182260.1	A
			Aythya fuligula	XM_032194046.1	A
			Numida meleagris	XM_021409943.1	A
			Meleagris gallopavo	XM_003210211.4	A
			Coturnix japonica	XM_015874845.2	A
			Gallus gallus	NM_001030606.1	A
			Phasianus colchicus	XM_031609429.1	A
			Eurypyga helias	XM_010151766.1	A
			Grus americana	KM508488.1	A
			Mesitornis unicolor	XM_010188920.1	A
			Tauraco erythrolophus	XM_009984861.1	A
			Opisthocomus hoazin	XM_009939978.1	A
			Acanthisitta chloris	XM_009080508.1	A
			Corvus brachyrhynchos	XM_008634462.2	A
			Corvus macrorhynchos	AB555651.1	A
			Corvus moneduloides	XM_032121195.1	A
			Corvus cornix cornix	XM_010390291.3	A
			Ficedula albicollis	XM_005053322.1	A
			Cyanistes caeruleus	XM_023935048.1	A
			Parus major	XM_015640791.1	A
			Pseudopodoces humilis	XM_005521947.1	A
			Zonotrichia albicollis	KM977611.1	A
			Lonchura striata domestica	XM_021542529.2	A
			Taeniopygia guttata	NM_001076695.1	A
			Serinus canaria	AJ277926.1	A
			Passerina cyanea	KM977612.1	A
			Emberiza bruniceps	JQ695942.1	A
			Cardellina pusilla	KM977610.1	A
			Geothlypis philadelphia	KM977605.1	A
			Geothlypis trichas	KM977604.1	A
			Mniotilta varia	KM977606.1	A
			Oreothlypis ruficapilla	KM977609.1	A
			Seiurus aurocapilla	KM977607.1	A
			Setophaga caerulescens	KM977596.1	A
			Setophaga castanea	KM977597.1	A
			Setophaga fusca	KM977598.1	A
			Setophaga magnolia	KM977599.1	A
			Setophaga palmarum	KM977600.1	A
			Setophaga pensylvanica	KM977601.1	A
			Setophaga petechia	KM977602.1	A
			Setophaga ruticilla	KM977608.1	A
			Setophaga striata	KM977603.1	A
			Corapipo altera	XM_027640753.1	A
			Lepidothrix coronata	XM_017827045.1	A
			Manacus vitellinus	XM_008926317.3	A
			Neopelma chrysocephalum	XM_027702634.1	A
			Pipra filicauda	XM_027746442.1	A
			Ailuroedus crassirostris	JQ034381.1	A
			Chlamydera maculata	JQ034385.1	A

	Chlamydera nuchalis	JQ034384.1	A
	Ptilonorhynchus violaceus	JQ034383.1	A
	Scenopoeetes dentirostris	JQ034380.1	A
	Sericulus chrysocephalus	JQ034382.1	A
	Rhipidura hypoxantha	KM977620.1	A
	Sturnus vulgaris	XM_014874082.1	A
	Abrornis chloronotus	KM977616.1	A
	Abrornis humei	KM977613.1	A
	Abrornis maculipennis	KM977614.1	A
	Phylloscopus occipitalis	KM977615.1	A
	Phylloscopus pulcher	KM977617.1	A
	Phylloscopus reguloides	KM977618.1	A
	Seicercus whistleri	KM977619.1	A
	Regulus regulus	KM977621.1	A
	Camarhynchus parvulus	XM_030956530.1	A
	Geospiza fortis	XM_005426641.1	A
	Empidonax traillii	XM_027903286.1	A
	Egretta garzetta	XM_009633823.1	A
	Pelecanus crispus	XM_009479489.1	A
	Phaethon lepturus	XM_010284327.1	A
	Phalacrocorax carbo	XM_009507226.1	A
	Nipponia nippon	XM_009469932.1	A
	Picoides pubescens	XM_009904062.1	A
	Strigops habroptila	XM_030500738.1	A
	Melopsittacus undulatus	AF021242.1	A
	Aptenodytes forsteri	XM_009283802.1	A
	Pygoscelis adeliae	XM_009320102.1	A
	Athene cunicularia	XM_026857140.1	A
	Apaloderma vittatum	XM_009875238.1	A
	Apteryx australis mantelli	XM_013954914.1	A
	Apteryx rowi	XM_026074997.1	A
	Dromaius novaehollandiae	KU568456.1	A
	Struthio camelus australis	XM_009681156.1	A
	Nothoprocta perdicaria	XM_026052520.1	A
	Tinamus guttatus	XM_010222333.1	A
	Chelonia mydas	XM_007059947.1	A
	Terrapene carolina triunguis	XM_024207554.1	A
testudines	*Chrysemys picta bellii*	XM_008168043.1	A
	Gopherus evgoodei	XM_030570330.1	A
	Pelodiscus sinensis	XM_006132837.3	A

Taxonomy	Species	accession numbers	RH1 292
	Amphisbaena sp.	KR336728.1	A
	Amphisbaena alba	KR336729.1	A
	Amphisbaena infraorbitale	KR336730.1	A
	Podarcis muralis	XM_028719287.1	A
	Takydromus sexlineatus	KR336727.1	A
	Bachia flavescens	KR336731.1	A
	Ophiodes striatus	KR336732.1	A
	Pogona vitticeps	XM_020813194.1	A
	Anolis carolinensis	NM_001291387.1	A
	Uta stansburiana	DQ100323.1	A
	Acrochordus javanicus	KX237831.1	A
	Ahaetulla nasuta	KX237852.1	S
	Arizona elegans	KU324006.1	S
	Bogertophis subocularis	KX237844.1	S
	Boiga forsteni	KX237867.1	A
	Cemophora coccinea	MH011456.1	S
	Chironius bicarinatus	MG544945.1	S
	Chironius carinatus	KX237846.1	S
	Chironius fuscus	KX237847.1	S
	Chrysopelea ornata	KX237851.1	S
	Dasypeltis scabra	KX237856.1	A
	Elaphe climacophora	KX237845.1	S
	Orthriophis taeniurus	KX237862.1	S
	Hemorrhois hippocrepis	KX237835.1	A
	Lampropeltis californiae	KU324004.1	A
	Lampropeltis getula floridana	KX237857.1	A
	Lampropeltis getula	MH011458.1	S
	Lycodon aulicus	KX237875.1	A
	Opheodrys aestivus	KX237839.1	S
	Pantherophis guttatus	KX237863.1	A
	Pseustes poecilonotus	KR336741.1	S

			Pituophis catenifer	KX237854.1	S
			Pituophis melanoleucus	MF076667.1	S
			Spalerosophis diadema	KX237853.1	S
			Spilotes pullatus	MG544957.1	S
			Telescopus fallax	KU324005.1	A
			Amphiesma stolatum	KX237866.1	S
			Natriciteres sylvatica	KX237833.1	A
			Natrix maura	KU324002.1	S
			Thamnophis elegans	XM_032212219.1	S
			Thamnophis proximus	KU306726.1	S
			Thamnophis sirtalis	MH011461.1	S
			Xenochrophis piscator	KX237865.1	S
			Atractus badius	KX237842.1	A
			Atractus flammigerus	KR336740.1	A
			Atractus reticulatus	MG544944.1	A
			Dipsas catesbyi	KX237848.1	A
			Dipsas indica	KX237849.1	A
			Sibynomorphus mikanii	MG544955.1	A
			Sibynomorphus neuwiedi	MG544956.1	A
			Echinanthera cephalostriata	MG544947.1	S
			Echinanthera undulata	MG544948.1	S
			Erythrolamprus aesculapii	MG544949.1	S
			Erythrolamprus miliaris	MG544950.1	S
			Erythrolamprus poecilogyrus	MG544951.1	S
			Erythrolamprus reginae	KX237855.1	S
			Helicops angulatus	KX237836.1	A
Sauropsida	squamata		*Helicops modestus*	MG544952.1	S
			Heterodon nasicus	KX237850.1	S
			Hypsiglena jani	KU324007.1	A
			Hypsiglena torquata	MH011457.1	A
		Serpentes (Snakes)	*Imantodes lentiferus*	KX237841.1	A
			Leptodeira annulata	KX237840.1	A
			Oxyrhopus guibei	MG544953.1	A
			Oxyrhopus melanogenys	KX237838.1	A
			Philodryas patagoniensis	MG544954.1	S
			Pseudoboa coronata	KX237837.1	A
			Sibon nebulatus	KX237843.1	A
			Taeniophallus persimilis	MG544958.1	S
			Thamnodynastes hypoconia	MG544959.1	S
			Thamnodynastes pallidus	KX237864.1	S
			Tomodon dorsatus	MG544960.1	S
			Xenopholis scalaris	KX237834.1	A
			Notechis scutatus	KU324000.1	A
			Pseudonaja textilis	XM_026722881.1	S
			Naja kaouthia	KX237830.1	A
			Ophiophagus hannah	PRJNA201683	S
			Sinomicrurus japonicus boettg	LC543598	S
			Laticauda colubrina	LC543602	S
			Laticauda frontalis	LC543601	S
			Laticauda laticaudata	LC543600	S
			Laticauda semifasciata	LC543599	S
			Enhydris innominata	KX237832.1	A
			Emydocephalus ijimae	LC543603	A
			Hydrophis melanocephalus	LC543605	A
			Hydrophis ornatus	LC543606	A
			Hydrophis peronii	KU324001.1	A
			Hydrophis platurus	LC543604	A
			Polemon collaris	KR336739.1	S
			Boaedon olivaceus	KX237859.1	A
			Gonionotophis sp.	KX237861.1	A
			Lycophidion laterale	KX237860.1	A
			Pareas monticola	KX237868.1	A
			Bothrops atrox	MH244492.1	A
			Bothrops jararaca	MK582578.1	A
			Crotalus durissus terrificus	MK582581.1	A
			Protobothrops mucrosquamatu	XM_015823472.1	A
			Bitis nasicornis	KX237873.1	A
			Causus rhombeatus	KX237876.1	A
			Echis ocellatus	KX237874.1	A
			Xylophis captaini	KX237869.1	S
			Anilius scytale	KR336736.1	A
			Eryx conicus	KX237870.1	A
			Python bivittatus	XM_007423262.1	A

	Python regius	FJ497236.1	A
	Tropidophis feicki	KR336738.1	A
	Melanophidium khairei	KX237871.1	A
	Uropeltis macrolepis	KX237872.1	A
	Xenopeltis unicolor	FJ497233.1	A
	Liotyphlops beui	KR336734.1	A
	Typhlophis squamosus	KR336733.1	A
	Epictia collaris	KR336735.1	A
	Amerotyphlops brongersmianu	KR336737.1	A
	Feylinia sp.	KR336742.1	A
	Melanoseps occidentalis	KR336743.1	A

Taxonomy	Species	accession numbers	RH1 292
	Amblysomus hottentotus	GQ290298.1	A
	Chrysochloris asiatica	XM_006868670.1	A
	Elephantulus edwardii	XM_006901040.1	A
	Macroscelides proboscideus	GQ290310.1	A
	Loxodonta africana	NM_001280858.1	A
	Trichechus manatus	AF055319.1	A
	Trichechus manatus latirostris	XM_004368355.1	A
	Echinops telfairi	XM_004702378.1	A
	Orycteropus afer afer	XM_007956743.1	A
	Galeopterus variegatus	XM_008574046.1	A
	Oryctolagus cuniculus	NM_001082349.1	A
	Ochotona princeps	XM_004581320.1	A
	Castor canadensis	XM_020175614.1	A
	Dipodomys ordii	XM_013010425.1	A
	Octodon degus	XM_004645617.3	A
	Bathyergus suillus	GQ290300.1	A
	Fukomys damarensis	XM_010637946.1	A
	Heliophobius argenteocinereus	GQ290306.1	S
	Heterocephalus glaber	XM_004870461.2	A
	Cavia porcellus	NM_001173085.1	A
	Chinchilla lanigera	XM_005387085.2	A
	Thryonomys swinderianus	GQ290319.1	A
	Jaculus jaculus	XM_004651581.1	A
	Cricetulus griseus	NM_001244407.1	A
	Mesocricetus auratus	XM_005066100.3	A
	Peromyscus leucopus	XM_028864082.1	A
	Peromyscus maniculatus bairc	XM_006978532.1	A
	Meriones unguiculatus	XM_021641029.1	A
	Grammomys surdaster	XM_028753569.1	A
	Mastomys coucha	XM_031380945.1	A
	Mus pahari	XM_021190968.1	A
	Mus caroli	XM_021165399.1	A
	Mus musculus	NM_145383.2	A
	Rattus norvegicus	NM_033441.1	A
	Nannospalax ehrenbergi	AF309568.1	A
	Microtus ochrogaster	XM_005365058.3	A
	Nannospalax galili	XM_008854920.1	A
	Sciurus carolinensis	LR738609.1	A
	Sciurus vulgaris	LR738630.1	A
	Ictidomys tridecemlineatus	XM_005333784.3	A
	Marmota flaviventris	XM_027931952.1	A
	Marmota marmota marmota	XM_015495128.1	A
	Urocitellus parryii	XM_026383100.1	A
	Cercocebus atys	XM_012060295.1	A
	Chlorocebus sabaeus	XM_007985378.1	A
	Macaca fascicularis	NM_001283360.1	A
	Macaca mulatta	XM_001094250.3	A
	Macaca nemestrina	XM_011734526.1	A
	Mandrillus leucophaeus	XM_011974200.1	A
	Papio anubis	XM_003906878.4	A
	Theropithecus gelada	XM_025377237.1	A
	Colobus angolensis palliatus	XM_011950692.1	A
	Piliocolobus tephrosceles	XM_023215965.2	A
	Rhinopithecus bieti	XM_017885138.1	A
	Rhinopithecus roxellana	XM_010381505.2	A
	Gorilla gorilla gorilla	XM_004036292.3	A
	Homo sapiens	NM_000539.3	A
	Pan paniscus	XM_003829435.3	A
	Pan troglodytes	XM_516740.7	A
	Pongo abelii	XM_002813145.4	A

	Hylobates moloch	XM_032149832.1	A
	Nomascus leucogenys	XM_003265030.4	A
	Aotus nancymaae	XM_012472901.2	A
	Callithrix jacchus	XM_008982215.2	A
	Cebus capucinus imitator	XM_017505088.1	A
	Sapajus apella	XM_032262385.1	A
	Saimiri boliviensis boliviensis	XM_003926159.2	A
	Carlito syrichta	XM_008047820.2	A
	Microcebus murinus	XM_012785306.2	A
	Propithecus coquereli	XM_012655172.1	A
	Otolemur crassicaudatus	AB112591.1	A
	Otolemur garnettii	XM_003796229.3	A
	Tupaia chinensis	XM_006160664.1	A
	Sus scrofa	NM_214221.1	A
	Camelus ferus	XM_006180073.2	A
	Hippopotamus amphibius	KC676966.1	A
	Balaena mysticetus	KC676958.1	A
	Balaenoptera acutorostrata	KC676959.1	S
	Balaenoptera musculus	KC676960.1	S
	Balaenoptera physalus	KC676961.1	S
	Megaptera novaeangliae	KC676969.1	S
	Eschrichtius robustus	KC676965.1	A
	Caperea marginata	KC676963.1	S
	Tursiops truncatus	NM_001280659.1	S
	Inia geoffrensis	KC676967.1	A
	Neophocaena phocaenoides	KC676970.1	S
	Phocoena phocoena	KC676971.1	S
	Phocoenoides dalli	KC676972.1	S
	Kogia breviceps	KC676968.1	S
	Platanista minor	KC676974.1	S
	Pontoporia blainvillei	KC676975.1	A
	Berardius bairdii	KC676962.1	S
	Tasmacetus shepherdi	KC676976.1	S
	Ziphius cavirostris	KC676977.1	S
	Canis lupus dingo	XM_025449310.1	A
	Canis lupus familiaris	NM_001008276.1	A
	Vulpes vulpes	XM_026016915.1	A
	Enhydra lutris	AY883931.1	A
	Enhydra lutris kenyoni	XM_022498107.1	A
	Lutra lutra	LR738421.1	A
	Mustela erminea	XM_032360881.1	A
	Mustela putorius furo	XM_004738577.1	A
	Odobenus rosmarus	AY883925.1	A
	Odobenus rosmarus divergens	XM_004395657.1	A
	Callorhinus ursinus	XM_025885151.1	A
	Eumetopias jubatus	XM_028101668.1	A
	Zalophus californianus	XM_027584573.1	A
	Erignathus barbatus	AY883932.1	A
	Hydrurga leptonyx	AY883930.1	A
	Leptonychotes weddellii	XM_006740235.1	A
	Mirounga angustirostris	AY228452.1	S
	Neomonachus schauinslandi	XM_021695166.1	A
	Phoca groenlandica	AF055318.1	A
	Phoca vitulina	AF055317.1	A
	Pusa hispida	AY883927.1	A
	Ailuropoda melanoleuca	XM_002921249.3	A
	Ursus arctos horribilis	XM_026501415.1	A
Mammalia	Ursus maritimus	XM_008697847.1	A
	Acinonyx jubatus	XM_015074629.2	A
	Felis catus	NM_001009242.1	A
	Puma concolor	XM_025920840.1	A
	Panthera pardus	XM_019433593.1	A
	Panthera tigris altaica	XM_007075434.1	A
	Suricata suricatta	XM_029917599.1	A
	Eubalaena glacialis	JQ730751.1	A
	Balaenoptera acutorostrata sc	XM_007192608.1	S
	Delphinus delphis	AF055314.1	S
	Globicephala melas	AF055315.1	S
	Lagenorhynchus obliquidens	XM_027093955.1	S
	Orcinus orca	XM_004284305.2	S
	Lipotes vexillifer	XM_007461564.1	A
	Delphinapterus leucas	XM_022562745.1	A
	Monodon monoceros	XM_029243393.1	A

Neophocaena asiaeorientalis a	XM_024756446.1	S
Physeter catodon	XM_007126220.3	S
Mesoplodon bidens	AF055316.1	S
Bison bison bison	XM_010862448.1	A
Bos mutus	XM_005902834.1	A
Bos taurus	NM_001014890.2	A
Bubalus bubalis	XM_006078900.2	A
Tragelaphus eurycerus	GU246485.1	A
Capra hircus	XM_018066700.1	A
Ovis aries	XM_004018534.4	A
Odocoileus virginianus texanus	XM_020872574.1	A
Camelus bactrianus	XM_010953086.1	A
Camelus dromedarius	XM_010984900.2	A
Vicugna pacos	XM_006206787.2	A
Eonycteris spelaea	GQ290305.1	A
Cynopterus brachyotis	GQ290303.1	A
Cynopterus sphinx	GQ863420.1	A
Dobsonia viridis	GQ290304.1	A
Nyctimene cephalotes	GQ290313.1	A
Pteropus alecto	XM_006917646.1	A
Pteropus giganteus	GU246487.1	A
Pteropus vampyrus	XM_011370659.1	A
Rousettus aegyptiacus	XM_016122023.1	A
Rousettus leschenaultii	GQ290317.1	A
Craseonycteris thonglongyai	GU246489.1	A
Saccopteryx bilineata	MK209460.1	A
Saccopteryx leptura	MK209461.1	A
Taphozous melanopogon	GQ290318.1	A
Hipposideros armiger	XM_019646023.1	A
Hipposideros commersoni	GU246488.1	A
Hipposideros larvatus	GQ290308.1	A
Hipposideros pomona	GQ863426.1	A
Hipposideros pratti	GQ290309.1	A
Megaderma spasma	GQ290316.1	A
Chaerephon plicatus	GQ290301.1	A
Molossus molossus	MK209465.1	A
Tadarida brasiliensis	MK209464.1	A
Mormoops blainvillei	MG873067.1	A
Pteronotus parnellii	MG873068.1	A
Pteronotus pusillus	MK209468.1	A
Pteronotus quadridens	MG873069.1	A
Chilonatalus micropus	MK209463.1	A
Noctilio leporinus	MK209472.1	A
Brachyphylla nana pumila	MK209482.1	A
Carollia brevicauda	MK209490.1	A
Carollia perspicillata	MK209489.1	A
Rhinophylla fischerae	MK209487.1	A
Rhinophylla pumilio	MK209488.1	A
Desmodus rotundus	MK209474.1	A
Anoura geoffroyi	MK209480.1	A
Glossophaga soricina	MK209481.1	A
Monophyllus redmani	MG873065.1	A
Lionycteris spurrelli	MK209485.1	A
Erophylla bombifrons	MK209484.1	A
Phyllonycteris poeyi	MK209483.1	A
Gardnerycteris crenulatum	MK209476.1	A
Macrotus waterhousii	MG873066.1	A
Phyllostomus discolor	XM_028518094.1	A
Phyllostomus elongatus	MK209478.1	A
Phyllostomus hastatus	MK209477.1	A
Tonatia saurophila	MK209475.1	A
Artibeus fraterculus	MK209500.1	A
Artibeus jamaicensis	MK209495.1	A
Artibeus planirostris	MK209499.1	A
Chiroderma villosum	MK209504.1	A
Dermanura bogotensis	MK209501.1	A
Mesophylla macconnelli	MK209486.1	A
Phyllops falcatus	MG873064.1	A
Sturnira ludovici	MK209493.1	A
Sturnira tildae	MK209494.1	A
Uroderma bilobatum	MK209492.1	A
Vampyrodes caraccioli	MK209491.1	A
Rhinolophus ferrumequinum	GQ290314.1	A

Rhinolophus pearsonii	GQ863432.1	A
Rhinolophus pusillus	GQ290315.1	A
Rhinolophus sinicus	GQ863434.1	A
Eptesicus fuscus	XM_008152292.2	A
Miniopterus fuliginosus	GQ290311.1	A
Miniopterus natalensis	XM_016209965.1	A
Myotis brandtii	XM_005870029.2	A
Myotis davidii	XM_006758300.2	A
Myotis laniger	GQ863435.1	A
Myotis lucifugus	XM_006083811.3	A
Myotis nigricans	MK209462.1	A
Myotis ricketti	GQ290312.1	A
Nyctalus plancyi	GQ863422.1	A
Erinaceus europaeus	XM_007517079.1	A
Sorex araneus	XM_004613232.1	A
Condylura cristata	XM_004692360.2	A
Equus asinus	XM_014838425.1	A
Equus caballus	XM_023619934.1	A
Equus przewalskii	XM_008531022.1	A
Ceratotherium simum simum	XM_004442424.2	A
Manis javanica	XM_017647349.1	A
Dasypus novemcinctus	XM_004477246.2	A
Sarcophilus harrisii	XM_003762449.2	A
Sminthopsis crassicaudata	AY159786.2	A
Caluromys philander	AY313946.1	A
Monodelphis domestica	XM_001366188.2	A
Phascolarctos cinereus	XM_020980962.1	A
Vombatus ursinus	XM_027864063.1	A
Ornithorhynchus anatinus	NM_001127627.1	A
Tachyglossus aculeatus	JX103830.1	A

RH1 with 292 S from aquatic and terrestrial species are shown in blue and gray, respectively.
