# Supplementary material for: Visual adaptation of opsin genes to the aquatic environment in sea snakes
Source: BMC Evol Biol. 2020 Nov 26;20:158. doi: 10.1186/s12862-020-01725-1 (PMC7690139; doi:10.1186/s12862-020-01725-1)
Supplement: Supplementary file 8 — Additional file 8: Table S4. Primer sequences. [file 12862_2020_1725_MOESM8_ESM.pdf]

Table S4. primer sequences

Amplification for cDNA

Primer name	Sequence 5' - 3'
sLWS_F1	GAAAGAAAGAGAACGTGGGAGAGA
sLWS_F2	AGCGCTGGGTTGTTGTTTGC
sLWS_R1	GATATGCATAGGGAATCCATATGCT
sRH1_F1	AGCAGTGTTAGCTTGGAAGGA
sRH1_F2	GGCTGTAGAAAGATACGTAGTAG
sRH1_R1	CCAGGTGAATTGAAAGTGGTATG
sSWS1_F1	TCTTCCAGAAGGGTCCACACT
sSWS1_F2	CTGGAGCAGGTTCCTCCCGGAAGGA
sSWS1_R1	TTGGTSRCGTTCCAACSTGYAGAG
sSWS1_R2	TTGTATTTGGTGCCCACCGTGTA

Amplification for cloning into pCMV vector and mutagenesis

F_LWS_pCMV	CACACAAAGCTTGCCACCATGACAGAGGCCTGGAATGTG
F_RH1_pCMV	CACACAAAGCTTGCCACCATGAATGGAACGGAAGGCCTTA
R_LWS_pCMV	TGTGTGGGTACCTGCCGGTGATACAGAAGAGTTAG
R_RH1_pCMV_ss	TGTGTGGGTACCCGCAGGGGAAACCTGACTT
R_RH2_pCMV_ts	TGTGTGGGTACCTGCAGGGGAAACCTGACTT
A292P_F	TCTGCCACCTTTCTTTGCTAAGAGCTCT
A292P_R	AAGAAAGGTGGCAGAGTCATGAAGATGG
E181H_F	CATCCCTCATGGCATGCAATGTTCATGC
E181H_R	GCATGCCATGAGGGATGTACCTGGACCA
E181N_F	CATCCCTAACGGCATGCAATGTTCATGC
E181N_R	GCATGCCGTTAGGGATGTACCTGGACCA

Amplification for genomic DNA

Primer name	Sequence 5' - 3'
LWS_F	AGGAGACAGACAAATGTCAATGAG
LWS_R	GTTGTAAAGGTTTCAGTATAACCA
RH1_F	GAAATGCTCCCAAGATATCTGA
RH1_R	TTCTGCTGGTTTCCCCCAATCTTGT
LWSe34	CACTTCTATAAAAGTCCTCTGGCT
sLWSe1_R	CCAAGGTTGAAGTCAGCTGAAGA
sLWSe2_F	ACAAGAATAGGGACTTTGTCTT
sLWSe2_R	AGAGAAAAGGAGCAGCATGATG
sLWSe3_R	GGAAAGCTGCTGAAAATGATGAGAA
sLWSe4_F	TCTTATCTTGGTCAGAATGTTGTGT
sLWSe4_R	GGAAGAAAGTGTGCTTTATAAGGA
sLWSe6_F	GGGATTATGTTGTATGTGGTGGAA
sRH1e1_R	TAAAGAACATGCCAAGAACTCTAC
sRH1e2_F	GGATCACAACAATGGAAATCAG
sRH1e2_R	CCCATTAGCTCCTTTAGCTCCAT
sRH1e5_F	TGGTTATTGCAGCTTATTCCTGAT
SWS1e1_F	AGCCGTTAAGCGGACTTTGTC
SWS1e1_R	CACACCTCTTAAAACAAGTG
SWS1e2_F	TGCAATTGTCCACTTCTAATTCCA
SWS1e2_R	TTCCTCTCTGTGCCCATCA
SWS1e3_F	GCTGGACTAGAAGACCTCCAA
SWS1e3_R	AAGAAAGGTCCTAAAACGGGGTGAA
SWS1e4_F	TTCACGACCCAACTGTGGGTTT
SWS1e4_R	TTCGTGTCAGCTTCTCTGACTTCA
SWS1e5_F	CCTTCCAGCTCTGCTATTCTGTA
SWS1e5_R	TTGGCATAGTCATCATCTTGGT
