# Supplementary material for: Visual adaptation of opsin genes to the aquatic environment in sea snakes
Source: BMC Evol Biol. 2020 Nov 26;20:158. doi: 10.1186/s12862-020-01725-1 (PMC7690139; doi:10.1186/s12862-020-01725-1)
Supplement: Supplementary file 1 — Additional file 1: Figure S1. Alignments of (a) LWS, (b) RH1, and (c) SWS1 amino acid sequences. [file 12862_2020_1725_MOESM1_ESM.pdf]

(a)

Amino acid position	6	17	23	24	25	28	40	51	54	60	64	69	96	103	114	119	124	134	139	167	177	179	180	181	196	224	228	233	284	285	297	304	305	307
S. j.boettgeri	N	N	K	E	T	A	E	V	L	V	V	V	L	V	F	I	M	A	A	D	I	S	C	A	H	I	A	V	T	F	A	S	I	A
O. hannah	.	D	R	.	.	.	.	.	I	.	.	.	.	.	.	.	.	.	.	.	.	.	.	.	.	.	.	.	.	.	.	.	.	.
L. semifasciata	.	D	.	Q	.	V	.	.	.	I	I	.	.	.	.	F	L	.	.	.	.	A	.	S	.	.	.	I	.	.	.	.	L	S
L. laticaudata	.	D	.	Q	S	V	A	.	.	I	I	A	.	L	.	F	L	.	.	.	.	A	.	S	.	.	.	I	A	.	.	.	L	S
L. frontalis	.	D	.	Q	S	V	.	.	.	I	I	.	.	.	.	F	L	.	.	.	.	A	.	S	.	.	V	I	.	.	P	.	.	P
L. colubrina	.	D	.	Q	S	V	.	.	.	I	I	.	.	.	.	F	L	.	.	.	.	A	.	S	.	.	.	I	.	.	P	.	.	P
N. scutatus	.	D	.	.	.	.	.	I	.	I	I	.	I	.	V	.	.	.	.	.	.	.	.	T	.	.	.	.	.	.	.	A	L	.
E. ijimae	.	D	.	.	.	.	.	.	.	I	.	.	I	.	I	.	L	V	.	G	.	.	A	I	.	.	.	.	.	.	.	A	L	.
H. platurus	.	D	.	.	.	T	.	.	.	I	.	.	I	.	I	.	.	V	.	G	M	A	S	F	H/N	.	A/V	.	A	S	.	A	L	S
H. melanocephalus	.	D	.	.	.	.	.	.	.	I	.	.	I	.	V	.	.	V	G	G	.	.	S	I	.	T	.	.	.	.	.	A	L	.
H. peronii	D	D	.	.	.	.	.	.	.	I	.	.	I	.	V	.	.	V	G	G	.	.	S	I	.	.	.	.	.	.	.	A	L	.
H. ornatus	.	D	.	.	.	.	.	.	.	I	.	.	I	.	V	.	.	V	G	G	.	.	S	I	.	.	.	.	.	.	.	A	L	.
Amino acid position in bovine RH1																						164			181				269					292

(b)

Amino acid position	11	19	35	36	60	81	85	104	133	146	150	154	155	160	173	189	198	213	217	218	225	239	253	292	300	304	311
S. j.boettgeri	I	I	W	K	Y	I	F	V	V	F	E	I	M	T	I	V	V	V	T	V	R	E	M	S	I	V	K
O. hannah	V	.	.	.	.	V	.	.	.	.	.	.	.	.	.	.	.	I	.	.	.	.	.	.	.	.	.
L. semifasciata	V	.	.	.	.	V	.	.	.	.	.	.	.	.	.	.	.	.	.	.	.	.	.	.	.	.	.
L. laticaudata	V	.	.	.	.	V	.	.	.	.	.	.	.	.	.	.	.	.	.	.	.	.	.	.	.	.	.
L. frontalis	.	.	.	.	.	V	.	.	I	.	.	.	.	.	.	.	.	.	.	.	.	.	.	.	.	.	.
L. colbrina	.	.	.	.	.	V	.	.	.	.	.	.	.	.	.	.	.	.	.	.	.	.	.	.	.	.	.
N. scutatus	V	.	C/W	Q	F	.	F/L	.	.	F/L	Q	V	I	S/T	.	.	.	.	I	I	.	.	I/M	A	V	.	R
E. ijimae	V	V	.	.	F	.	.	.	.	.	Q	V	.	.	V	I	.	I	I	I	.	.	.	A	V	.	.
H. platurus	V	V	.	Q	F	.	.	.	.	.	Q	.	.	.	V	.	I	I	I	I	.	.	.	A	V	.	.
H. melanocephalus	V	V	.	Q	F	.	.	.	.	.	Q	.	.	.	V	.	I	I	I	I	.	.	.	A	V	.	.
H. peronii	V	V	.	Q	F	.	.	I	.	.	Q	.	.	.	V	I	I	I	I	I	S	G	.	A	V	M	.
H. ornatus	V	V	.	Q	F	.	.	.	.	.	Q	.	.	.	V	.	I	I	I	I	.	.	.	A	V	.	.
Amino acid position in bovine RH1																								292			

(c)

Amino acid position	3	5	6	8	10	11	12	14	19	38	44	52	57	70	80	82	87	88	89	101	104	107	108	120	129	150	154	161	164	205	210	221	258	259	276	278	311	314	321	322	323	328	329	338	346	347
O. hannah	E	E	D	F	L	Y	E	I	P	I	F	A	V	F	L	F	V	F	V	L	Q	K	M	T	F	L	V	V	S	V	I	R	C	I	Q	G	A	M	P	M	T	V	A	A	K	T
S. j.boettgeri	-	-	-	-	-	-	-	-	-	L	.	.	I	.	.	.	.	.	T	F	.	.	L	.	.	.	A	.	G	.	V	.	.	.	.	.	.	L	A	.	.	.	T	.	.	S
L. semifasciata	.	.	E	.	.	F	K	.	.	.	.	T	.	.	.	.	.	.	.	F	.	.	L	.	.	.	A	.	.	I	.	Q	.	T	.	N	.	.	.	.	M	.	.	V	.	S
L. laticaudata	.	.	E	Y	.	F	.	.	.	.	.	T	I	.	.	.	.	.	.	F	.	R	L	.	.	.	A	.	A	I	.	.	.	T	.	N	.	.	.	.	M	.	T	V	.	S
L. frontalis	.	.	E	Y	.	F	.	.	.	.	.	T	.	.	.	.	.	.	.	F	.	R	L	.	.	.	A	.	A	I	.	.	.	T	H	S	.	.	.	.	M	.	.	V	.	S
L. corbrina	.	.	E	Y	.	F	.	.	.	.	.	T	.	.	.	.	.	.	.	F	.	R	L	.	.	.	A	.	A	I	.	.	.	T	H	S	.	.	.	.	M	.	.	V	.	S
N. scutatus	G	.	-	.	.	.	K	T	T	.	.	.	I	S	.	.	.	.	.	F	.	.	L	.	V	.	A	.	G	.	.	.	.	T	.	.	G	.	.	.	M	.	V	V	-	-
E. ijimae	.	-	-	.	I	.	.	.	T	.	L	.	I	.	F	S	I	S	.	F	L	.	L	I	.	.	A	I	G	I	.	.	G	M	H	.	G	.	S	K	.	.	V	V	.	S
H. platurus	.	D	-	.	I	.	.	.	T	.	.	.	I	.	F	.	.	.	.	F	.	.	L	.	.	.	A	.	A	I	.	.	G	.	.	.	G	.	.	.	M	M	V	V	.	S
H. melanocephalus	.	D	-	.	I	.	.	.	T	.	.	.	I	.	F	.	.	.	.	F	.	.	L	.	.	.	A	.	A	I	.	.	G	.	.	.	G	.	.	.	M	.	V	V	E	S
H. peronii	G	.	-	.	.	.	.	.	T	.	.	.	I	.	F	.	.	.	.	F	.	.	L	.	.	.	A	.	A	I	.	.	G	.	.	.	G	.	.	.	M	.	V	V	-	-
H. ornatus	-	-	-	.	I	.	.	.	T	.	.	.	I	.	F	Y	.	.	.	F	.	.	L	.	.	.	A	.	A	I	.	.	G	.	.	.	G	.	.	.	M	.	V	V	.	S
Amino acid position in bovine RH1																85																														

Fig. S1 Alignments of (a) LWS, (b) RH1, and (c) SWS1 amino acid sequences
